# Professor Guislau

**Published:** 1860-07

**Authors:** 


PROFESSOR GUISLAU, author of several works on the physiology and diseases of the nervous system, and the Director of the Ghent Asylum, died recently of strangulated hernia, for which an operation had been performed. He bequeathed fifty thousand francs to the civil charities of Gand, and his library and pictures to the Insane Asylum. By royal decree, his bust in marble will be placed in the hall of the Academy of Medicine.

